# New insights into real-time detection of tephra grainsize, settling velocity and sedimentation rate

**DOI:** 10.1038/s41598-022-08711-1

**Published:** 2022-03-17

**Authors:** V. Freret-Lorgeril, C. Bonadonna, E. Rossi, A. P. Poulidis, M. Iguchi

**Affiliations:** 1 Department of Earth Sciences, University of Geneva, 13, Rue des Maraichers, CH-1205 Geneva, Switzerland; 2 Disaster Prevention Research Institute (DPRI), Kyoto University, Kagoshima, Japan; 3 Institute of Environmental Physics (IUP), University of Bremen, Bremen, Germany

**Keywords:** Volcanology, Natural hazards

## Abstract

Characterizing the size and settling velocity of pyroclastic fragments injected into the atmosphere during volcanic eruptions (i.e., tephra) is crucial to the forecasting of plume and cloud dispersal. Optical disdrometers have been integrated into volcano monitoring networks worldwide in order to best constrain these parameters in real time. Nonetheless, their accuracy during tephra fallout still needs to be assessed. A significant complication is the occurrence of particle aggregates that modify size and velocity distributions of falling tephra. We made the first use of the Thies Clima Laser Precipitation Monitor (LPM) for tephra-fallout detection at Sakurajima volcano (Japan), which is characterized by a lower size detection window with respect to more commonly used disdrometers (e.g., Parsivel^2^) and can more easily distinguish different falling objects. For the first time, individual particles have been distinguished from most aggregates based on disdrometer data, with the potential to provide useful grain-size information in real time. In case of negligible aggregation, LPM and collected sample-based estimates are in agreement for both grain-size and sedimentation rate. In case of significant aggregation, particle shape analyses and a dedicated drag equation are used to filter out aggregates from LPM data that also provide good agreement with collected tephra samples.

## Introduction

The determination of the total grain-size distribution (TGSD) of volcanic particles emitted during explosive eruptions, i.e., tephra, is essential for volcanic plume monitoring and modelling and for the mitigation of risks associated with tephra dispersal and deposition^1–3^. However, among all Eruptive Source Parameters (ESPs) that are used to initialize dispersal models, such as mass eruption rate and volcanic plume height, the TGSD is the most difficult to determine in real time. Typically, the TGSD is determined after the end of an eruptive event based on the combination of the grain-size distribution (GSD) of individual locations/outcrops of a tephra-fallout deposit^4^.

During the last two decades, weather-dedicated radar and optical disdrometers have been introduced in volcanology to determine the size and velocity of tephra falling at various volcanoes such as Etna^5^, Eyjafjallajökull^6^, Sakurajima^7–11^ and Stromboli^12–14^. The optical disdrometers have the advantage of determining size and velocity simultaneously, allowing for a better characterization of tephra particles, whereas radar disdrometers require additional backscatter models to provide particle sizes^5,6,15^. These optical instruments have been tested under various field conditions and represent one of the most promising techniques to determine GSDs and ground accumulation in real time, based on direct measurements of settling particles. Disdrometers have also been used to cross-validate remote sensor observations of volcanic plumes, such as Doppler radar data^7–9,12,13^. However, no studies aiming at discriminating the different types of falling objects during volcanic fallout have been carried out so far, unlike for weather-dedicated systems (e.g., Fig. 9 of Löffler-Mang and Joss^16^).

A particular aspect that can affect tephra fallout is particle aggregation^17–24^. In fact, volcanic ash (tephra < 2 mm) has been shown to mostly fall as aggregates of various types ranging from particle clusters (ash clusters, coated particles and cored clusters) to accretionary pellets (poorly-structured pellets, concentric pellets and liquid pellets)^19,22^. Particle aggregation can clearly affect the size and velocity distributions of tephra detected by disdrometers in real time. In fact, particle aggregates can have diameters and velocities 1–2 orders of magnitude larger than most of the constituting particles^17^. If disdrometer data are to be used to provide GSDs in real time, an effort to distinguish individual particles and particle aggregates is crucial to avoid biased reconstructions of the TGSD.

**Figure 1:**
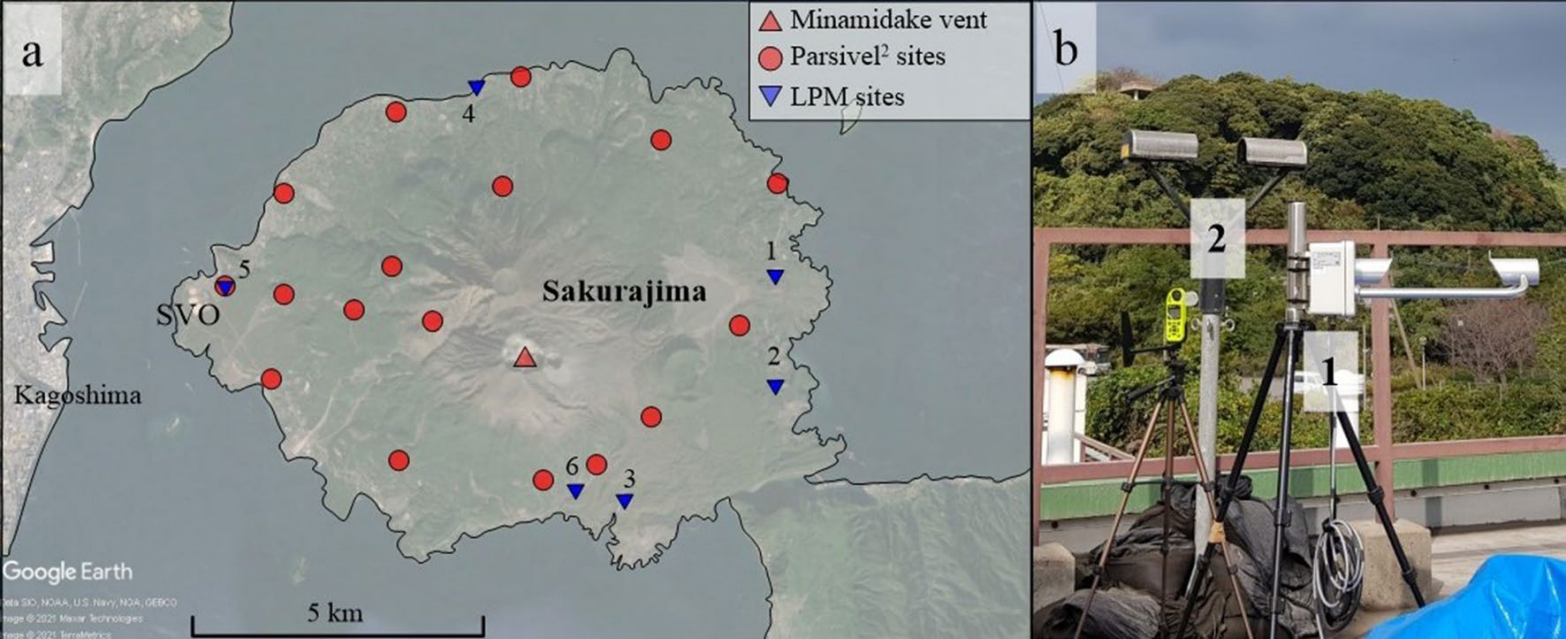
(**a**) Map of Sakurajima Volcano and disdrometer sampling sites (blue triangles: LPM sites; red dots: PS2 sites). SVO indicate the monitoring station from which PS2 data have been compared with LPM records. Numbers indicate the index of each site reported in Table 1. Map generated from Google Earth Pro 7.3.4.8248 available in https://www.google.fr/earth/download/gep/agree.html. (**b**) Picture of the LPM (1) next to the PS2 (2) located on the roof of the SVO (Sakurajima Volcano Observatory) (Photograph taken by V. Freret-Lorgeril).

In this study, we take advantage of a new optical disdrometer never used before in volcanology for characterizing tephra fallout, the Thies Clima Laser Precipitation Monitor (called LPM hereafter). As the OTT Parsivel^2^ (called PS2 hereafter), the relatively low-cost LPM (i.e., currently twice cheaper than PS2 with prices < 4 kUSD) is one of the most used optical disdrometer for meteorological studies^25^ and monitoring liquid and solid hydrometeor precipitation. The PS2 has been increasingly used in volcanology to monitor tephra fallout^7–9,11^; however, in addition to the low cost that could increase the potential number of instruments deployed in the field, the LPM has some additional advantages in comparison to PS2. Firstly, data acquired on each measured particle can be accessed (i.e., “drop by drop” mode; see “Materials and methods” section). Secondly, it has a lower size detection (i.e. 0.15 mm vs. 0.25 mm^25–27^. The improved observation capabilities of the LPM have the potential to facilitate the discrimination of individual particles and particle aggregates during fallout. We tested the LPM at Sakurajima volcano (Japan), one of the most active volcanoes worldwide that has been erupting frequently since 1955^28^ and whose fallout is frequently characterized by the occurrence of volcanic aggregates^22,29–33^. A network of 12 PS2 disdrometers (at the time of the field campaign; Fig. 1a,b) has been installed around the Sakurajima peninsula since 2017 to monitor tephra fallout^11,22,31,32,34^. Our main objective is to explore the potential of LPM to provide the size distribution of individual volcanic particles in real time and mitigate the impact of particle aggregation. Disdrometer records are here compared with data obtained independently on falling aggregates during the same measurement campaign by Diaz-Vecino et al.^33^.

**Table 1:** Eruption and tephra-fallout information. Time is expressed in local time (i.e., UTC + 9).

Date	Eruption time	Sample index	Fallout beginning time	Fallout Duration (s)	SITE (Lat/long)	Tray	Ground accumulation (× 10^–3^ kg m^−2^)
15/11/2019	14:29:20	II*	14:48:15	485	1 (31.592/130.708)	1	16.46
15/11/2019	17:09:54	III*	17:27:51	375	2 (31.574/130.708)	1	6.10
16/11/2019	10:10:29	IV	10:28:10	1890	3 (31.555/130.679)	1	5.71
16/11/2019	11:33:17	VI	11:46:10	1434	3 (31.555/130.679)	2	7.64
16/11/2019	13:49:34	VII*	13:57:00	1160	3 (31.555/130.679)	2	21.36
16/11/2019	14:06:07 + 14:09:34 + 14:21:55 + 15:13:02	VIII	14:23:36	4584	3 (31.555/130.679)	1	38.32
16/11/2019	14:06:07 + 14:09:34 + 14:21:55	VIII-1	14:37:36	1885	3 (31.555/130.679)	2	22.82
16/11/2019	15:13:02	VIII-2	15:14:30	1025	3 (31.555/130.679)	2	20.96
16/11/2019	15:31:02 + 15:36:50	XI	15:43:44	1383	3 (31.555/130.679)	1	21.11
16/11/2019	16:05:44	X*	16:20:52	778	3 (31.555/130.679)	1	12.56
17/11/2019	13:22:35	XII*	13:38:32	550	4 (31.623/130.650)	1	8.50
17/11/2019	13:51:50	XIII*	14:04:12	1085	4 (31.623/130.650)	1	142.78
23/11/2019	16:11:00	XIV*	14:28:20	1665	5 (31.590/130.601)	1	315.11
25/11/2019	10:16:15	XVI	10:21:41	828	6 (31.557/130.669)	2	68.28

*Samples characterized also by laser diffraction by Diaz-Vecino et al.^33^.

## Results

### Characterization of tephra samples collected on ground

In total, we collected tephra samples during 14 tephra-fallout events that were also recorded by the LPM (Table 1). Sample total mass accumulation was between 5.7 and 315.1 × 10^–3^ kg m^−2^. Tephra samples were manually sieved down to 0.5 mm; the fraction < 0.5 mm was analyzed for grain-size and shape using the microscopy option of the BETTERSIZER (BTS) morpho-grainsizer. The size distributions of both sieved fractions and analyzed material were then combined. The resulting grain-size distributions (GSD_BTS_ hereafter) are very well sorted with sorting coefficients *σ*_*Φ*_^35^ comprised between 0.37 and 1.62, while *Md*_*Φ*_^35^ ranges between 0.63 (i.e., 0.65 mm) and 3.21 (i.e., 0.11 mm) (see Table 2).

**Table 2:** Grain-size parameters as derived from both tephra samples (whole and partial) and LPM data.

Sample index	Whole sample (GSD_BTS_)			Partial sample (i.e., GSD_BTS_ > 0.15 mm)					LPM raw data			LPM no “margin fallers”				LPM individual particles			
	Md_Φ_	σ_Φ_	SR (kg m^2^ s^−1^)	Md_Φ_	σ_Φ_	Mean Cv	Mean Sd	Mean Ψ	Md_Φ_	σ_Φ_	Particle Number	Md_Φ_	σ_Φ_	Particle Number	SR (kg m^2^ s^−1^)	Md_Φ_	σ_Φ_	Particle Number	SR (kg m^2^ s^−1^)
II*	1.50	0.37	3.39E−5	1.49	0.37	0.95 ± 0.01	0.92 ± 0.05	0.63 ± 0.07	1.42	0.36	2014	1.40	0.35	1848	4.84E−5	1.43	0.35	1740	4.31E−5
III*	1.46	0.39	1.63E−5	1.45	0.38	0.95 ± 0.01	0.92 ± 0.03	0.63 ± 0.06	1.44	0.36	859	1.43	0.35	780	1.20E−5	1.45	0.35	748	1.11E−5
IV	1.31	0.37	3.02E−6	1.22	0.28	0.95 ± 0.01	0.93 ± 0.02	0.65 ± 0.06	1.24	0.63	737	1.21	0.63	634	1.33E−5	1.31	0.31	595	5.43 E−6
VI	1.40	0.41	5.33E−6	1.39	0.40	0.95 ± 0.02	0.93 ± 0.03	0.64 ± 0.06	1.45	0.38	1271	1.41	0.36	1050	0.99E−5	1.44	0.36	973	8.84E−6
VII*	1.47	0.47	1.18E−5	1.46	0.46	0.95 ± 0.02	0.92 ± 0.04	0.63 ± 0.06	1.57	0.46	4595	1.51	0.40	3572	2.35E−5	1.52	0.41	3031	1.96 E−5
VIII	1.79	0.51	8.36E−6	1.76	0.49	0.95 ± 0.02	0.91 ± 0.04	0.62 ± 0.06	1.78	0.39	14,882	1.71	0.32	10,067	1.69E−5	1.73	0.31	8728	1.41 E−5
VIII-1	1.78	0.56	7.84E−6	1.75	0.53	0.95 ± 0.02	0.91 ± 0.04	0.62 ± 0.06	1.76	0.42	5244	1.69	0.35	3607	1.51E−5	1.71	0.34	3004	1.20 E−5
VIII-2	1.86	0.46	1.32E−5	1.86	0.44	0.95 ± 0.02	0.91 ± 0.04	0.62 ± 0.06	1.81	0.37	5163	1.73	0.30	3386	2.39E−5	1.75	0.27	3004	2.02 E−5
XI	1.62	0.43	1.53E−5	1.62	0.42	0.95 ± 0.02	0.92 ± 0.03	0.63 ± 0.06	1.73	0.36	6631	1.68	0.33	4893	2.97E−5	1.70	0.31	4296	2.49 E−5
X*	1.44	0.41	1.61E−5	1.43	0.40	0.95 ± 0.02	0.92 ± 0.04	0.06 ± 0.06	1.59	0.39	3050	1.57	0.37	2378	3.32E−5	1.56	0.37	2196	2.98 E−5
XII*	2.39	1.44	1.55E−5	1.47	0.54	0.95 ± 0.02	0.90 ± 0.06	0.61 ± 0.08	0.22	0.56	444	0.22	0.56	433	3.88E−5	1.09	0.36	103	2.46E−6
XIII*	1.34	1.15	1.32E−4	1.23	0.50	0.94 ± 0.02	0.90 ± 0.05	0.63 ± 0.07	0.85	0.48	8049	0.85	0.47	7702	2.29E−4	1.01	0.4	5908	1.39E−4
XIV*	3.21	1.14	1.89E−4	1.97	0.79	0.94 ± 0.02	0.87 ± 0.06	0.59 ± 0.08	-0.39	0.96	5947	-0.41	0.95	4579	2.89E−4	0.46	0.66	3014	8.76E−5
XVI	0.63	1.62	8.25E−5	0.60	0.87	0.94 ± 0.02	0.87 ± 0.06	0.60 ± 0.08	0.33	0.82	4385	0.31	0.79	3386	1.89E−4	0.19	0.85	2008	9.96E−5

“Raw data”: LPM data without any filter. “No margin fallers”: LPM data without particles falling above the margin trend (see Fig. 3).

“Individual particles”: LPM data without margin fallers and without particles falling below the individual-particle trend (see main text and Fig. 3 for more details).

*Samples also analyzed by Diaz-Vecino et al.^33^.

Additionally, all GSDs_BTS_ were recomputed without taking into account material below 0.15 mm (i.e., the LPM lower detection limit) in order to compare them with their respective GSDs_LPM_ (i.e., grain-size distributions obtained in real time with LPM). All GSDs_BTS_ associated with the fraction > 0.15 mm (defined as partial samples hereafter) are also well sorted (*σ*_*Φ*_ between 0.37 and 0.87). All *Md*_*Φ*_ values are similar to the values associated with the total samples with the exception of events XII and XIV that have values of 1.47 (0.36 mm) *vs* 2.39 (0.19 mm) and 1.97 (0.26 mm) *vs* 3.21 (0.11 mm), respectively for the partial and the total samples (Table 2). All sample GSDs_BTS_ can be seen in Fig. S1 of the Supplementary Material. In addition, all acronyms and symbols used in this study are shown in the Supplementary Material 1.

**Figure 2:**
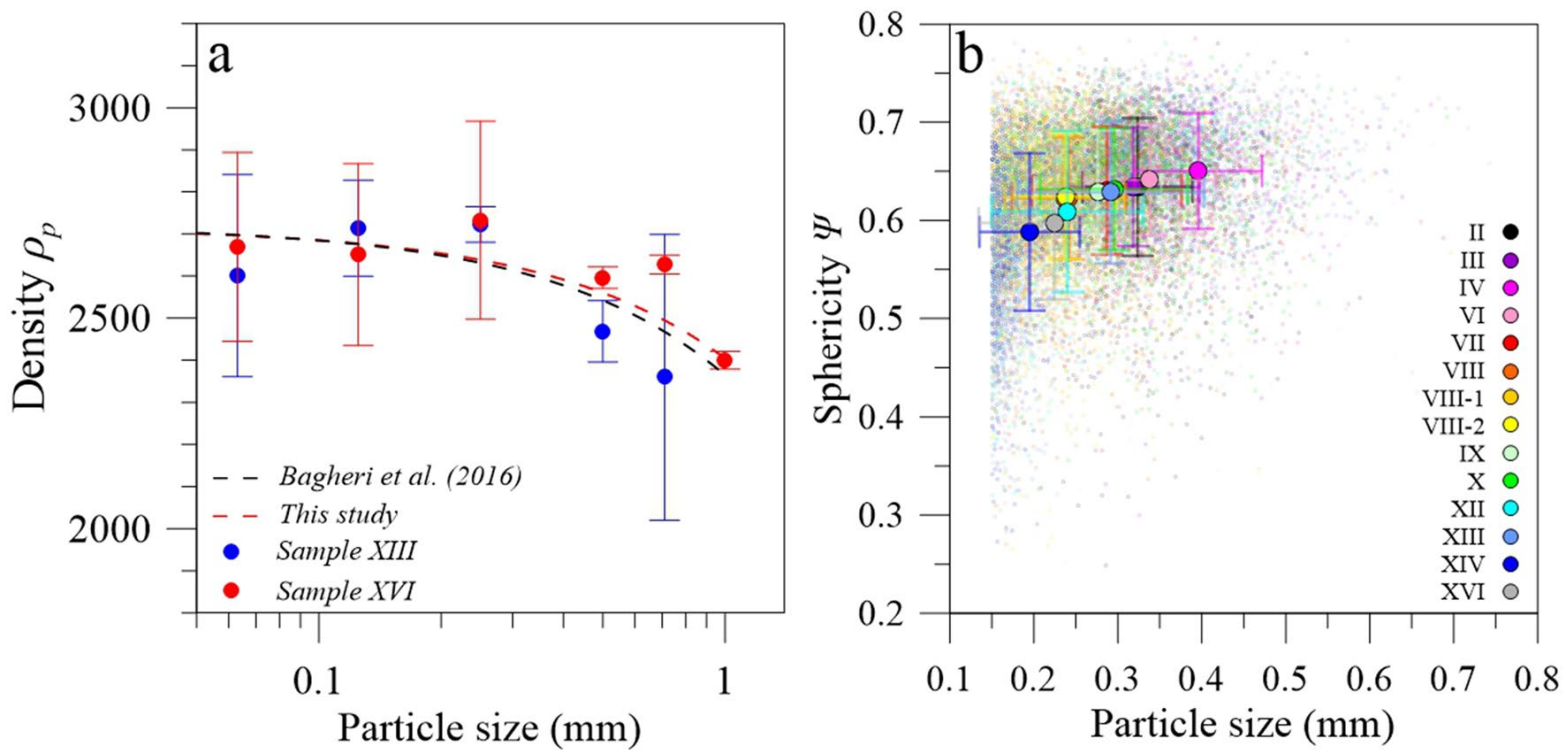
(**a**) Semi-log plot of particle densities ρ_p_ (kg m^−3^) determined by water pycnometry as a function of particle sizes (mm). Blue and red dots correspond to particles from samples XIII and XVI, respectively. Black and red dashed lines corresponds to density best fits obtained by Bagheri et al.^22^ and considering our data, respectively (see main text for more details). (**b**) Particle sphericities Ψ^36^ determined with the BTS for all analyzed fractions of each tephra sample (small colored dots). Larger circles and error bars indicate the mean sphericity of each analyzed sample and its standard deviation.

Values of particle convexity and solidity are very similar among all partial samples, i.e., particles > 0.15 mm, with mean values ranging between 0.94 ± 0.02 and 0.95 ± 0.01, and 0.87 ± 0.06 and 0.93 ± 0.02, respectively (Table 2). The density *ρ*_*p*_ of sieved material from samples XIII and XVI between 63 µm up to 1.0–1.4 mm was determined by water pycnometry. In particular, we observe high density values decreasing from ~ 2700 kg m^3^ to ~ 2400 kg m^-3^ with increasing sizes (Fig. 2b). Such values are similar to those obtained by Bagheri et al.^22^ for the whole analyzed size range. We also determine a density trend, similar to that of Bagheri et al.^22^, in the form *ρ*_*p*_(kg m^−3^) = 312 × *D*(mm) + 2716 with a relatively good R^2^ of 0.84. The aforementioned particle convexity and solidity are mainly > 0.80 and high densities are coherent with observations of dense and regular ash fragments^37^. In addition, values of particle sphericity *Ψ* are similar among all partial samples (Fig. 2b) and range between less than 0.30 up to 0.79 with an overall mean of 0.62 ± 0.06.

### Validation of the laser precipitation monitor for ash detection

#### Examples of LPM disdrometer records

**Figure 3:**
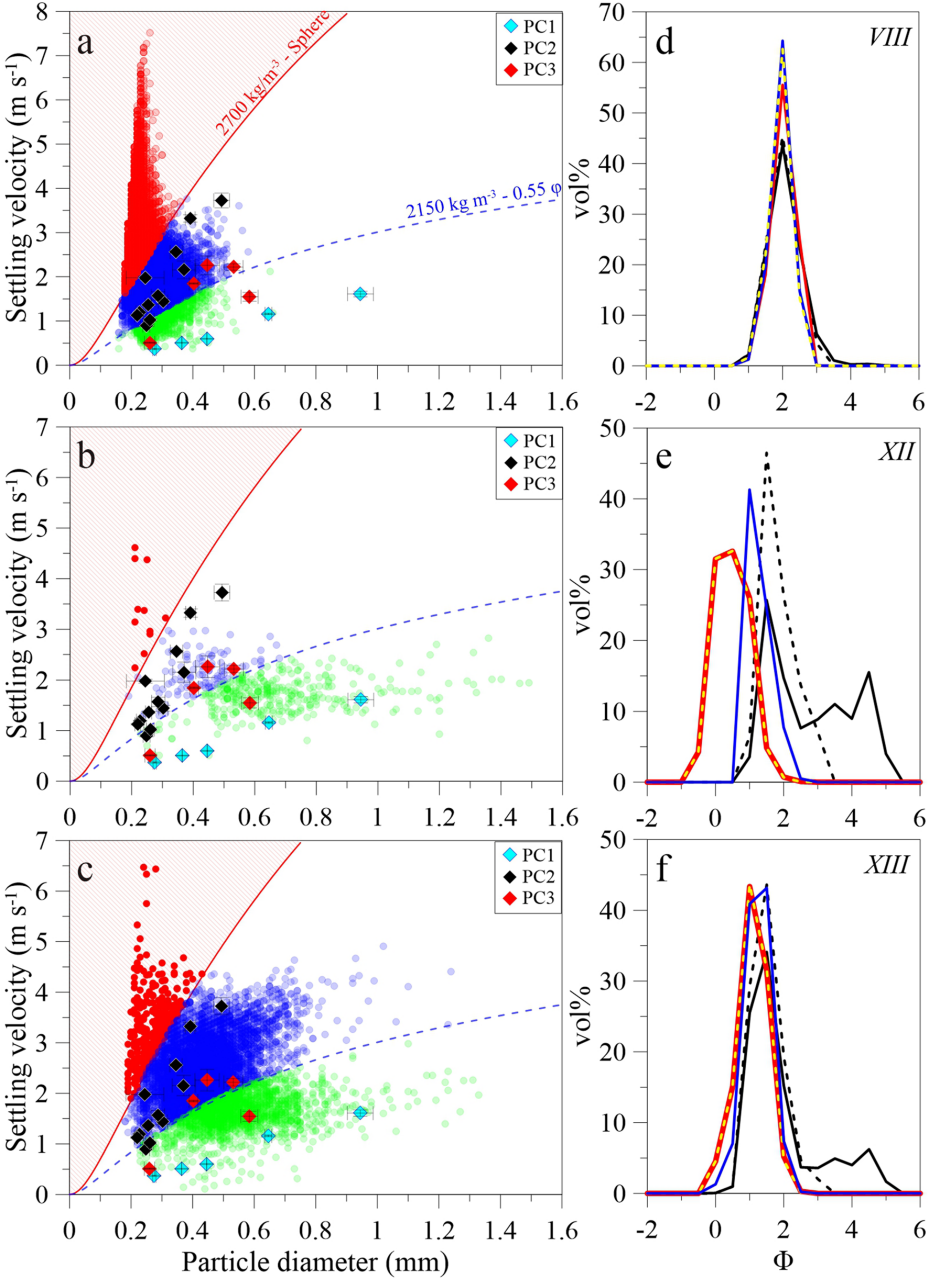
Particle size and velocity measured during the tephra-fallout events (**a**) VIII, (**b**) XII and (**c**) XIII. Red and blue dashed lines indicate the margin trend and the individual-particle trend, respectively. Diamonds indicate aggregate estimates retrieved based on high-speed camera analyses by Diaz-Vecino et al.^33^ (blue: Ash Cluster—PC1; black: Coated Particle—PC2; red: Cored Cluster—PC3). Vol% GSD of LPM records and obtained from BETTERSIZER analysis for events VIII, XII and XIII are shown in (**d**), (**e**) and (**f**), respectively. Red: raw LPM data; dashed yellow: LPM data without margin fallers; Blue: LPM filtered for single settling particles; Black: raw BETTERSIZER distributions; Dashed Black: GSDs_BTS_ of particles > 0.15 mm.

In order to investigate LPM data, we consider three tephra-fallout events associated with Vulcanian eruptions with plumes between 1.0 and 3.4 km high (a.s.l.). It is important to note that all hours in this study are in local time (i.e., UTC + 9). On 16 November 2019, we recorded three tephra-fallout events at disdrometer site 3 (Fig. 1a) between 14:20 and 15:40 (sample VIII; Table 1). The Particle Size and Velocity Distribution (PSVD) for this sequence displays 14,896 particles with velocities and sizes ranging from 0.38 to 7.51 m s^−1^ and 0.16 to 0.66 mm, respectively (Fig. 3a). On 17 November at 13:38 and 14:04, we observed two tephra-fallout events at site 4 (Fig. 1a) associated with two Vulcanian eruptions; these events resulted in PSVD of 445 particles (event XII) and 8049 particles (event XIII). While event XII was mainly composed of low velocity (0.48–4.61 m s^−1^) and large-size particles (0.19–1.49 mm) (Fig. 3b), the PSVD of event XIII presented velocity and size ranges of 0.11–6.47 m s^−1^ and 0.18–1.33 mm, respectively (Fig. 3c).

For all recorded tephra-fallout events, including events VIII, XII and XIII, PSVDs present a sub-vertical trend of high settling velocities corresponding to small sizes < 0.3 mm. Such a trend has already been observed in studies of hydrometeors and has been associated with particles crossing the edge of the laser beam and called “*margin fallers*”^25^ hereafter. First, such an incomplete detection of falling particle induces an underestimation of the particles crossing time, hence overestimating their settling velocities. Second, it induces smaller amplitudes of the laser extinction causing the retrieved particle sizes to be underestimated. In order to filter the data from these margin fallers*,* we use the drag equation of Ganser^38^ (see the “Materials and methods” section) assuming a maximum *Ψ* of 1 (spherical case) and a maximum density of 2700 kg m^−3^, as derived in Fig. 2a and by Bagheri et al.^22^ for particles between 0.06 and 0.15 mm. Such a set of particle parameters results in the red size/velocity trends in Fig. 3a–c, named *margin trend* hereafter. This filter is used because tephra are also non-spherical particles (e.g., *Ψ* < 1)^37,39,40^, hence, all data falling above the margin trend cannot be considered as valid, i.e., particles with *ρ*_*p*_ > 2700 kg m^−3^ and/or *Ψ* ≥ 1 (with *Ψ* > 1 being mathematically impossible). The GSD_LPM_ of events VIII, XII and XIII display *Md*_*Φ*_ values of 1.78, 0.22 and 1.95, respectively, and *σ*_*Φ*_ between 0.39 and 0.56 (Table 2). Importantly, filtering all particles above the margin trend, i.e., all margin fallers, has no or only minor effect on the retrieved GSDs_LPM_ from all recorded events including VIII, XII and XIII, with differences of 0.00–0.08 in *Md*_*Φ*_ and 0.00–0.07 in *σ*_*Φ*_ values (Table 2; Fig. 3d–f).

#### Comparison with tephra samples

**Figure 4:**
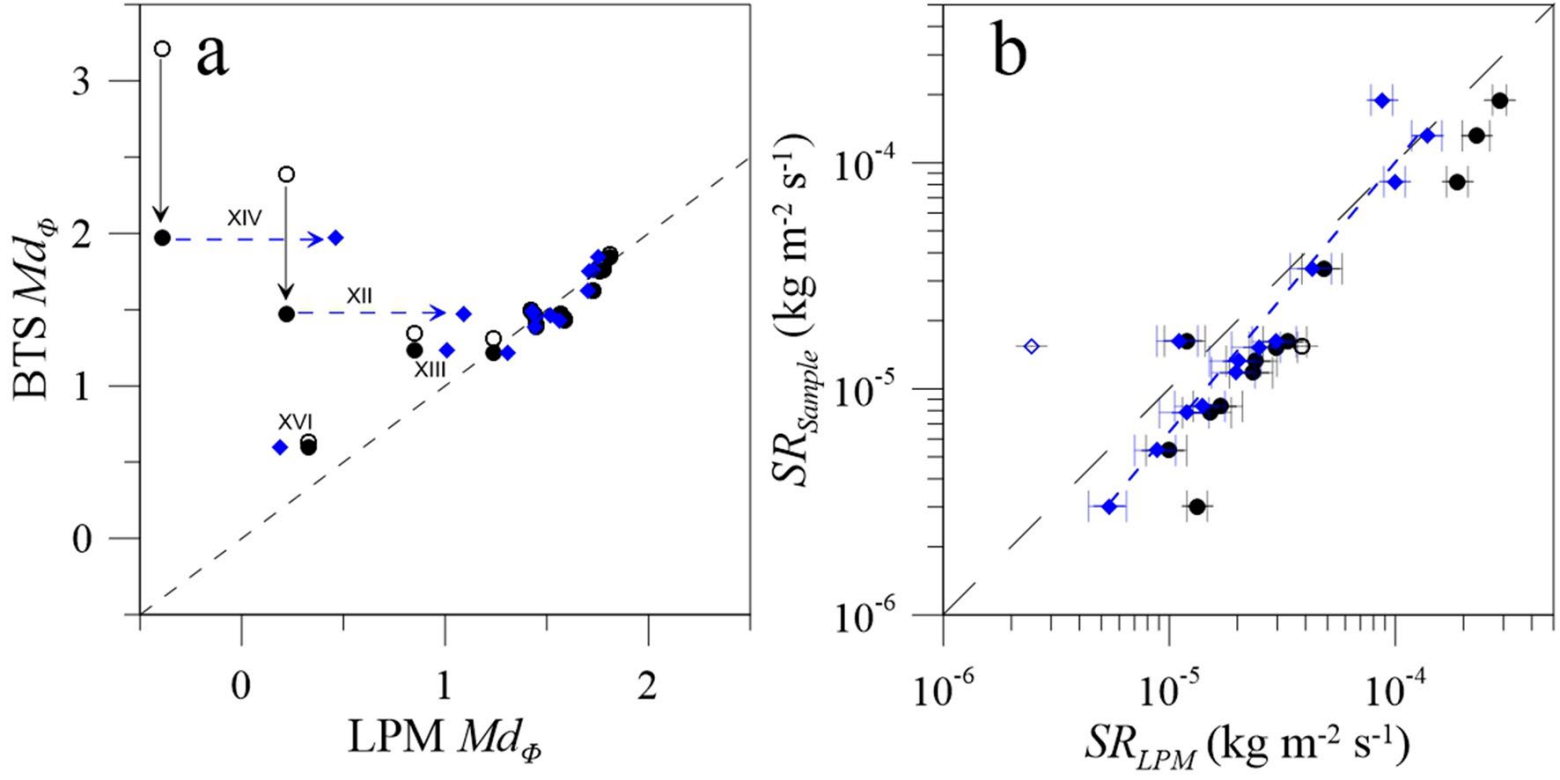
(**a**) Md_Φ_ of the GSDs_LPM_ from recorded tephra fallout as a function of Md_Φ_ values of the GSD_BTS_ based on collected samples. Open circles: raw GSDs_LPM_ (including margin fallers); Black dots: GSD_BTS_ for particles > 0.15 mm vs GSDs_LPM_ with margin faller filter; Blue diamonds: GSD_BTS_ for particles > 0.15 mm vs GSD_LPM_ of individual particles (i.e., applying both the margin faller and the individual-particle lowest velocity filters); Black and blue arrows indicate the effect on Md_Φ_ values of filtering particles below 0.15 mm in GSDs_BTS_ and filtering the aggregates in GSDs_LPM_, respectively. The black dashed line is the one-to-one line. (**b**) Sedimentation Rate in kg m^−2^ s^−1^ computed based on sampling in trays (SR_Sample_) and from the LPM records (SR_LPM_) of all fallout events. Black: LPM data without margin fallers; Blue diamonds: SR_LPM_ based on GSDs_LPM_ with both margin fallers and individual-particle lowest velocity filter; The open symbol indicates event XII for which a large quantity of aggregates was observed in the LPM records (see Fig. 3b). The dashed blue line corresponds to a power-law between SR_Sample_ and SR_LPM_ for individual particles (see text for more details). Horizontal error bars of the mean differences of SR_LPM_ for size classes whose width dD is ± 0.05 mm (see the “Materials and methods” section).

Among the 14 tephra samples collected at the same time as the LPM detection of fallout events, ten of them present GSDs_BTS_ that are fairly similar to the GSDs_LPM_ without applying any filter to either dataset (Table 2; Fig. 4a). In particular, these ten events display similar *Md*_*Φ*_ values with differences comprised between 0.01 and 0.15 Φ (Table 2). However, GSDs of events XII, XIII (Fig. 3e,f), XIV and XVI (Fig. S1) show larger discrepancies in *Md*_*Φ*_ values with maximum differences of 2.17 Φ (i.e., event XII) and 3.6 Φ (i.e., event XIV) (Fig. 4). For these four tephra-fallout events, GSDs_BTS_ are bimodal with coarse peaks between 0.5–1.5 Φ (i.e., 0.71 and 0.35 mm) and finer ones around 3.5–4.5 Φ (i.e., 0.09 and 0.04 mm) (Figs. 3d–f and S1). This bimodality was also observed in samples XII and XIII by laser diffraction^33^. Given that the LPM is not able to detect particles below 0.15 mm, GSDs_BTS_ have been filtered down to this limit in order to make them comparable to GSDs_LPM_ (black circles in Fig. 4). Such a filter brings successfully all *Md*_*Φ*_ values closer to the one-to-one line with respect to *Md*_*Φ*_ values of GSDs_LPM_, except for events XII and XIV whose remaining discrepancies are discussed hereafter.

Additionally, one of the main objectives of using optical disdrometers is to determine the particle sedimentation rate, which is a critical parameter to track ground mass loading in real time. We compare mean sedimentation rate *SR*_*Sample*_ obtained from collected samples with the sedimentation rate *SR*_*LPM*_ computed from LPM records in Fig. 4b (see Eq. 2 in the “Materials and methods” section). For each fallout event, particle densities are computed using our density trend obtained in Fig. 2a and *Md*_*Φ*_ values of each GSD_LPM_. Overall, we find that *SR*_*Sample*_ values are very low and comprised between 3.02 × 10^–6^ and 1.89 × 10^–4^ kg m^−2^ s^−1^. An additional interesting result is the linear increase from 0.99 ± 0.20 × 10^–5^ to 2.89 ± 0.21 × 10^–4^ kg m^−2^ s^−1^ observed in the log–log plot in Fig. 4b between *SR*_*Sample*_ and *SR*_*LPM*_ considering LPM data without margin fallers. Moreover, all sedimentation rates can be fitted by a power-law in the form *SR*_*Sample*_ = 0.62 × (*SR*_*LPM*_)^*1.01*^ with a good R^2^ of 0.90.

#### Comparison between LPM and PS2 records

**Figure 5:**
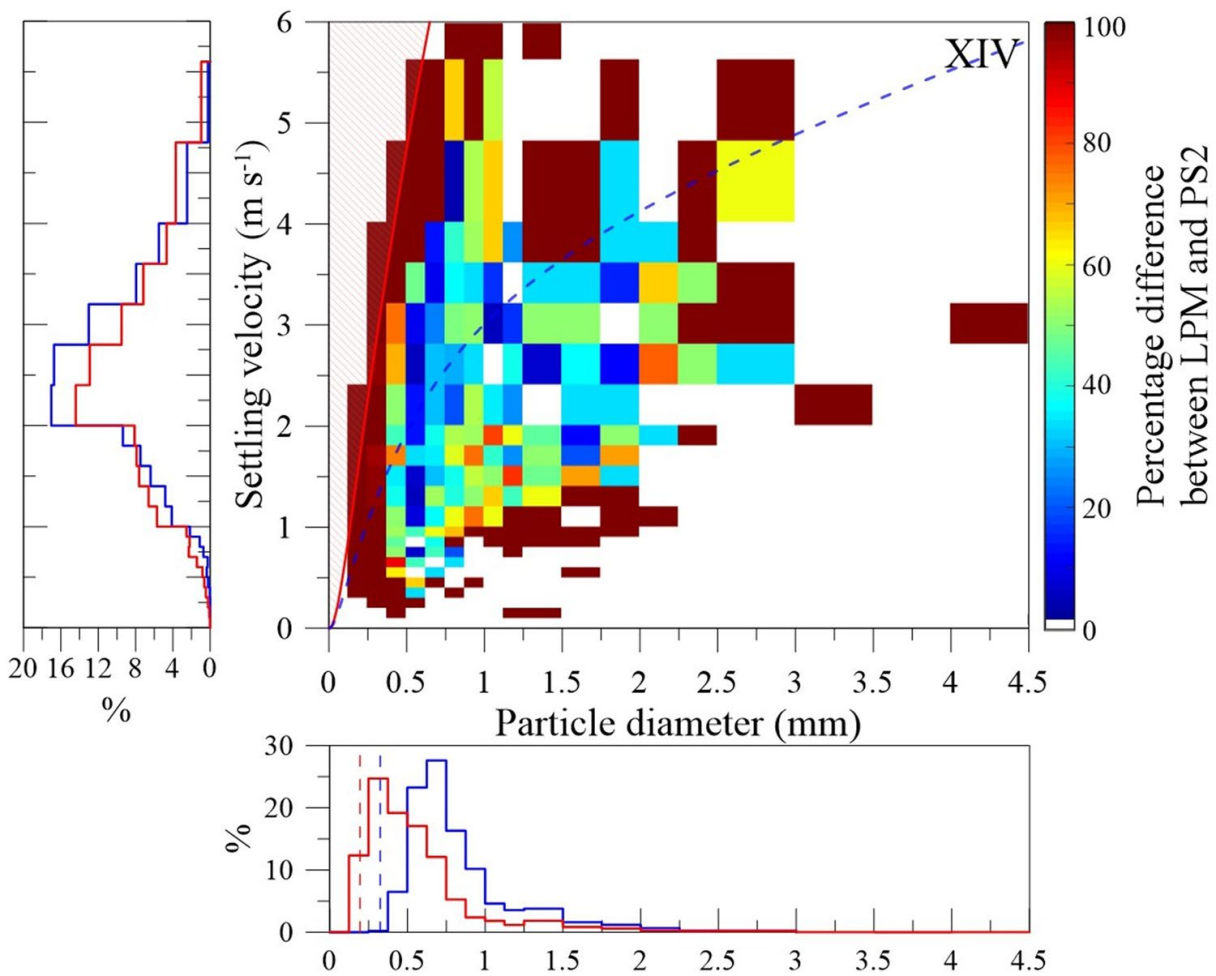
PSVD difference in percentage between LPM vs PS2 records of event XIV (station SVO in Fig. 1a). LPM data are reshaped following the size and velocity bins of PS2 data. Margin fallers have been corrected from LPM data. The red line indicates the trend used to filter margin fallers. The dashed blue line indicates the individual-particle trend. Left and down panels present the number percentage distributions of settling velocities and particle sizes using PS2 class widths, respectively (blue line: PS2; red line: LPM without margin fallers). All velocity and size distributions are in number frequency for a better comparison between both disdrometers. Vertical dashed blue and red lines in both down panels indicate the lower detection limits of the PS2 and the LPM, respectively.

One tephra-fallout event was monitored by both LPM and PS2 at the same location on the 23 November 2019 (event XIV in Tables 1 and 2). It is important to note that all LPM estimates listed in this section are provided once margin fallers have been filtered to make both GSDs comparable (such detections are corrected internally by the PS2^25^). During event XIV, we set up our LPM just next to the PS2 on the roof of the SVO (Fig. 1). PS2 detected less particles than the LPM with a total of 3035 against 5947 particles in LPM-based PSVDs. In fact, over 204 bins containing detected particles that are in common between both disdrometers, 40% present discrepancies of 100% and 34% show discrepancies < 50%. Nonetheless, settling velocities are in the same range between 0.4 and 4.8 m s^−1^ with particle sizes mainly ranging from 0.35 to 3 mm (Fig. 5).

In order to compare GSDs and Grain-Velocity Distributions (GVDs) of both disdrometers (see step curves in left and down panels of Fig. 5), we reshaped LPM data using each width of size and velocity classes used for PS2 records^7,12^ (see the “Materials and methods” section). Accordingly, the reshaped GSD_LPM_ of event XIV displays a lower median of 0.44 ± 0.13 mm against 0.69 ± 0.13 mm for GSD_PS2_. Interestingly, the GVDs of both disdrometers have similar mode of 2.2 ± 0.4 m s^−1^.

### Application of the LPM to discriminate individual tephra particles from aggregates

As stated before, the presence of particle aggregation significantly affects the real-time reconstruction of GSDs^31^ related to individual particles. Hence, in addition to the margin-faller filter, we apply a filter to exclude objects characterized by a terminal velocity that is lower than that associated with individual particles, which is associated with the lowest value of density (2150 kg m^−3^, i.e., density for a 1-mm particle^22^) and the lowest value of *Ψ* (~ 0.55, i.e., the value associated with 90% of particles; Fig. 2b). Such a set of parameters results in a trend called the *individual-particle trend* (e.g., blue dashed line in Fig. 3a–c). Hence, all particles that fall below this trend are assumed to not correspond to individual settling particles.

Our filtering strategy is in good agreement with observations of size and settling velocities obtained on falling aggregates by Diaz-Vecino et al.^33^ using a high-speed camera during the same measurement campaign. In fact, objects falling below the individual-particle trend in the LPM records form a trend which is in agreement with objects that are identified as ash clusters or PC1^19,29^. Both individual particles and those falling below the single settling trend form two clear trends that are fairly separated by the two filters (e.g., Fig. 3b,c). Assuming that particles falling below the single settling trend are mostly aggregates, the PSVD of event VIII presents 13% of aggregates while events XII and XIII are characterized by 76% and 23% of aggregates, respectively.

In the case of all events excluding XII, XIII, XIV and XVI, GSDs_LPM_ are associated with similar *Md*_*Φ*_ values whether the aggregates are filtered out or not (e.g., *Md*_*Φ*_ of 1.73 *vs* 1.71, for event VIII) (Table 1; Fig. 3d). Such events can be considered as individual-particle-dominated events. However, aggregates can significantly affect both GSDs associated with collected tephra samples and with LPM records. For events XII to XVI, the occurrence of a large amount of particles falling below the settling trend associated with the bimodality of the four GSDs_BTS_ (characterized by a secondary population of very fine material) is in agreement with the observation of falling aggregates made by Diaz-Vecino et al.^33^ during the same fallout events. Accordingly, GSDs_LPM_ are significantly different when the contribution of aggregates is high such as for fallout XII, e.g., *Md*_*Φ*_ of 1.09 *vs* 0.22 when filtering or not the aggregates (Table 1; Fig. 3e). GSDs_BTS_ of sample XII to XVI are richer in fine ash than GSDs_LPM_, especially in particles ≤ 40 microns (i.e., Figs. 3e,f and S1), which is due to the aggregate breakage when impacting the ground and during manual sieving^31,41,42^. In addition, all GSDs_LPM_ are offset towards larger sizes, e.g., events XII and XIV (Figs. 3 and S1).

In particular, the aforementioned observations are in agreement with observations of PC1 and two PC3 made during our campaign^33^ (Fig. 3b,c). Indeed, PC1 aggregates (ash clusters) are known to be associated with large sizes, low settling velocities and low densities^19,31^. PC3 aggregates (cored clusters) are characterized by an internal core, i.e., 0.20 to 0.70 mm single particles, covered by a shell of fine ash whose volume can vary significantly, affecting the sizes and aerodynamics of such aggregates^22,31^. This is why not all PC3 aggregates are filtered out by the single settling trend. Consequently, GSDs_BTS_ are similar to GSDs_LPM_ of individual particles once filtered down to 0.15 mm (blue diamonds in Fig. 4a), especially for event XII with *Md*_*Φ*_ values of 1.47 *vs* 1.09 (i.e., 0.36 *vs* 0.47 mm), respectively. The difference associated with filtered GSDs of event XIV will be discussed below (i.e., highest blue square in Fig. 4a).

Regarding the sedimentation rates, even though the *SR*_*LPM*_ values without margin fallers are slightly offset towards larger values than the one-to-one line in Fig. 4b, *SR*_*LPM*_ of individual particles (i.e., without margin fallers and aggregates) are in the same range than *SR*_*Sample*_ with values between 2.5 ± 0.4 × 10^–6^ and 1.4 ± 0.2 × 10^–4^ kg m^−2^ s^−1^. However, the filtered *SR*_*LPM*_ of event XII is underestimated (i.e., 2.5 ± 0.4 × 10^–6^ kg m^−2^ s^−1^ against an *SR*_*Sample*_ of 1.6 × 10^–5^ kg m^−2^ s^−1^). Nonetheless, without considering the latter event for which a large quantity of suggested aggregates was observed (e.g. 76%) (Fig. 3b), we obtain a power-law trend between all values that is closer to the one-to-one line and in the form *SR*_*Sample*_ = 5.98 × (*SR*_*LPM*_) ^*1.19*^ with a R^2^ of 0.91.

Finally, both disdrometer records display particles falling below the trend of individual particles (Fig. 5) with similar proportions, i.e., 34% and 30% respectively in LPM and PS2 data.

## Discussion

The discrimination between individual particles and aggregates is crucial to the determination of GSD in real time. In fact, GSD determined at individual locations that is used to obtain the TGSD in real time should only contain information on individual particles. We have shown how our disdrometer data filtering based on single settling particles can help to discriminate individual particles from aggregates (i.e., PC1 and most PC3 aggregates). In fact, the different aggregate types are characterized by different densities^31,33^, and, therefore, different aerodynamics behavior. Based on the individual-particle lowest density filter, we are not only able to filter PC1 (forming a linear trend) but also some PC3 aggregates which are more dispersed (Fig. 3), both aggregates types having a lower density than individual particles. In contrast, PC2 (coated particles) have similar density with respect to individual particles^31,33^, and, therefore, cannot be discriminated. In fact, most of PC2 fall above our individual-particle lowest velocity trend (Fig. 3a–c). Nonetheless, size and velocity of PC2 aggregates are representative of size and velocity of the core particle due to a very low amount of aggregated fine material^31^. Hence, they can be integrated in the GSD because they act like individual particles and have a low impact on the amount of fine ash in the collected samples. Therefore, we clearly show that the sedimentation rate computed from the LPM is not largely affected by the presence of PC2 (Fig. 4b), but most likely by PC1 aggregates as shown by event XII.

As shown above, GSDs_BTS_ of partial samples (i.e., fraction of particles > 0.15 mm) and the GSDs_LPM_ of individual particles have similar *Md*_*Φ*_ values for all events (Fig. 4a), except for event XIV which is an outlier with respect to the general trend with an overestimated GSD_BTS_*-*based *Md*_*Φ*_. This issue is not due to the presence of PC1 aggregates in the LPM records as they represent only 34% of the total number of particles detected, according to the suggested filter criterion; as an example, the event XII was associated with 76% of aggregates, but the application of the filter provides satisfying results (Fig. 4a). Instead, this large *Md*_*Φ*_ value associated with the event XIV might result from a sampling bias of the tephra analyzed by the BTS. Indeed, BTS analyses consist in measuring the size of particles contained in a small amount of material introduced in the morpho-grainsizer. While the analysis is repeated with several spoons of the same sample, the large amount of very fine material in sample XIV might have led to an underestimation of the quantity of coarse material represented by a few large particles (see Table 2 and Fig. S1).

In addition to the differences in size detection between the two types of disdrometers, the sedimentation rate from PS2 (*SR*_*PS2*_) of XIV is higher than *SR*_*LPM*_ without margin fallers. Indeed, considering a same detection duration of ~ 14 min between 14:28:45 and 14:42:45, we obtained a *SR*_*PS2*_ of 7.2 ± 0.1 × 10^–4^ kg m^−2^ s^−1^ against 2.9 ± 0.2 × 10^–4^ kg m^−2^ s^−1^ for the *SR*_*LPM*_ at the same sampling site. The value of *SR*_*PS2*_ was computed using a density of 2502 kg m^−3^ based on our density trend (Fig. 2a) and on the mode of the GSD_PS2_ (Fig. 5). However, *SR*_*LPM*_ is much closer to the rate retrieved in the sample XIV, i.e., 1.9 × 10^–4^ kg m^−2^ s^−1^.

It is important to keep in mind that we compare data from instruments with different detection windows. Hence, while differences in GSDs are systematically shown for the two instruments^25,27^, including for volcanic particles (Fig. 4), this is not the case for settling velocities. We show here that velocity distributions of both disdrometers are similar and share similar modal values for a strong tephra-fallout event (left panel in Fig. 5), i.e., with 4579 detected particles (without margin fallers) during 28 min by the LPM (Table 2). However, this comparison holds only if LPM data are filtered from margin fallers, also because such unwanted detections are supposedly corrected internally in PS2 records^25^. In addition, margin fallers have been typically observed under rainfall events and are most likely to be seen in LPM records than those from PS2s^25,43^ (Fig. 5). This detection issue is supposedly due to a combination of larger laser lengths but smaller areas for LPMs compared with those of PS2s, i.e., with laser sizes of 22.8 × 2 cm *vs* 18 × 3 cm^25^, increasing the probability of measuring more margin fallers than complete detections when the rate of falling material increases. The number of such detections has also been shown to increase with wind speed close to the ground^44^. Notwithstanding this issue, in the range of tephra sizes recorded during the campaign, filtering or not the margin fallers from the LPM records has no significant impact on the determination of the GSD_LPM_ as shown by the overlap of distributions in Figs. 3d–f and S1. The wind speed was also low during the campaign and mostly ranging between 1.5 and 3.6 m s^−1^ close to the ground during our operating days (i.e., values taken from Kagoshima radiosounding reports; http://weather.uwyo.edu/upperair/sounding.html). In addition, it is important to note that tephra particles are not spherical and their maximum density is often around 2700 kg m^−3^ for andesitic to basaltic compositions (e.g. Sakurajima^22^, Stromboli and Etna volcanoes in Italy^12,45,46^). Hence, margin fallers can easily be filtered from LPM records using the Ganser^38^ drag equation assuming densities of 2700 kg m^−3^ and a sphericity of 1 (Figs. 3a–c and 4). Finally, Tokay et al.^47^ and Angulo-Martínez et al.^25^ already discussed the fact that PS2 might underestimate the settling velocity of rain droplets compared to LPMs. Such an underestimation might probably impact records of volcanic particles as well. Hence, the difference of ~ 0.10 and 0.30 mm observed in GSD of event XIV between both disdrometers might be sufficiently small to make LPM and PS2-based GVDs similar thanks to the underestimated velocities in PS2 records (Fig. 5). In addition, this underestimation of velocities would lead to underestimate *SR* values. However, *SR*_*PS2*_ is still larger than *SR*_*LPM*_ because the GSD_PS2_ is coarser than that of our LPM and particle sizes have a larger impact on *SR* estimates than settling velocities (i.e., with an exponent of 3 in Eq. 2).

Our observations show that both LPM and PS2 present noticeable differences in the determination of particles sizes. This is mainly due to their respective characteristics. First, LPM is associated with a lower size detection with respect to PS2, providing data on particles down to 0.15 mm. Second, LPMs have been shown to provide higher numbers of detected hydrometeors than PS2^25,27^, in agreement with our observations of volcanic ash in Fig. 5, which is potentially due to a very limited detection sensitivity close to its lower detection limit. As an example, in the size class 0.25–0.36 mm, LPM detects 1132 particles against 6 for the PS2. In addition, LPM has been shown to provide higher numbers of detected hydrometeors than PS2 also when considering common ranges of particle sizes between 0.25 and 0.50 mm^25,27^. Indeed, starting from the same detection limit of 0.25 mm, the LPM detects 4014 particles against 3035 for the PS2. Both observations could be due to the fact that, close to their low detection limits, the number of particles detected in PS2 records is underestimated with very few ash particles detected in the first size-bin between 0.25 and 0.37 mm. Accordingly, GSDs_LPM_ are typically finer than GSDs_PS2_ even when considering the same range of detected tephra. While LPM and PS2 present complementary characteristics for ash monitoring, further investigations should aim at comparing both disdrometer estimates. Indeed, our measurements were performed under small tephra plumes produced by relatively weak Vulcanian explosions (max plume heights of about 2 km above sea level) and under similar weather conditions. This is why comparing both disdrometer estimates with other methods and for different eruptive/atmospheric conditions (e.g., under rainfall and/or extreme ash fallout) would help to test and verify their capacity for operational monitoring and the calibration of other remote sensing systems such as Doppler radar^8,9,12^ or dispersal models^10,11^.

Third, once margin fallers are filtered, LPM might be associated with less uncertainty in object detection. In fact, a typical detection bias observed for optical disdrometers consist in the measurement of simultaneous particles seen as one big object, i.e., double detection events. Indeed, when two particles cross the laser beam at the same time, disdrometers usually detect particles with over- and underestimated sizes and settling velocities, respectively. As shown by Angulo-Martínez et al.^25^, the probability of such detections increases with the laser-beam sampling area and should affect PS2 records more than LPM ones with their respective *S* of 0.0054 and ~ 0.0045 m^2^. With their expected coarse size/low velocity signatures, double-detected particles may lie below our individual-particle trend in Fig. 3a–c and be filtered, having no impact on our corrected estimates of grain-sizes and sedimentation rate. Moreover, GSDs_BTS_ and GSDs_LPM_ for individual-particle dominated events (i.e., without aggregation processes but during which double detections could still be measured) still present similar results with or without filtering the particles falling below the individual-particle trend. Hence, although the occurrence of double detections cannot be excluded, they do not seem to affect significantly our LPM records of tephra fallout, even in the case of a long sequence of ash sedimentation, i.e., event VIII with a duration of 76.4 min. These observations suggest that, unlike for particle velocity distributions in the case of margin fallers, expected detection bias typically observed for optical disdrometers such as margin fallers and double detections do not affect our LPM estimates of grain-sizes and sedimentation rates.

There is an increasing impact of the aggregates on the LPM estimates of sedimentation rates. With 23 to 41% of suggested aggregates in records of events XIII, XIV and XVI, there is a *SR*_*LPM*_ variation of 40 to 70% in between unfiltered and filtered data (see Table 2). For event XII, with 76% of aggregates, *SR*_*LPM*_ is biased towards smaller values (difference of 94%) when considering only individual particles. Interestingly, without filtering the data of the latter fallout, the *SR*_*LPM*_ is closer to *SR*_*Sample*_ (i.e., open circle in Fig. 4b). This means that, even in the case of fallout events with more than 50% of falling aggregates, LPM data can still be used in real time to determine sedimentation rates that are in the same order of magnitude than data collected on ground. In addition, it is important to note that small variations of particle densities may impact sedimentation rate estimates. As a matter of fact, we respectively find variations of 8.6% and 1.0% on *SR*_*LPM*_, with or without filtering LPM data, when using densities of Bagheri et al.^22^ or those retrieved from our samples (Fig. 2a), i.e., with variations of only 0.0–1.7% between both density trends.

Finally, both *SR* trends in Fig. 4b present a small bias compared to the one-to-one line, filtering or not both LPM and collected sample data. Such a small discrepancy in our dataset could be associated with the detection of non-spherical settling particles, which might fall with their largest and intermediate axes perpendicular to the direction of settling tending to overestimate the overall particle size when detected by the LPM^12,48^. Nonetheless, such an effect does not impact our record of particle size significantly, especially when comparing the grainsize distributions of both the disdrometer and the collected tephra samples (i.e., Fig. 4a).

## Conclusions

Disdrometers are essential tools for monitoring and characterizing tephra sedimentation in real time, especially in remote places and/or dangerous areas close to active volcanoes. However, some complications such as the occurrence of volcanic ash aggregates impact the accuracy of disdrometer detections. In this study, we made the first measurements of GSD, settling velocities and sedimentation rates of falling tephra at Sakurajima volcano using a Laser Precipitation Monitor and compared them with both tephra samples collected in dedicated trays and Parsivel^2^ disdrometers that are more commonly used for volcano monitoring. Based on these detailed comparisons, LPM has been shown to provide fundamental insights into GSD of individual particles at individual locations (during tephra fallout with and without particle aggregation) that can be combined to determine TGSD in real time, a key input parameter of Volcanic Ash Transport and Dispersal Models. Such a capability is mostly due to the use of “drop-by-drop” detection mode that can help discriminate falling tephra types (individual particles and ash aggregates that can significantly impact GSDs) through the application of dedicated filters (i.e., margin fallers and single-settling particles). LPM can also provide fundamental insights into rate of falling tephra even without the application of these filters. This shows the potential for the LPM to constrain near-ground tephra concentration and, hence, radar reflectivity factor data to help constrain Doppler radar observations. As a result, LPM retrievals associated with a critical data processing to eliminate the effect of particle aggregates represents a key new system that can effectively complement real-time monitoring of tephra dispersal and sedimentation at active volcanoes. In addition, our strategy for the detection of single particles and filtering of aggregates during tephra fallout can be applied for the processing of data associated with other types of disdrometers.

## Materials and methods

### Measurement campaign and Sakurajima activity

Sakurajima is an active stratovolcano located in the South part of Kyushu Island in Japan (Fig. 1a). It is one of the most active volcanoes worldwide with a persistent activity since 1955^28,49^. Its activity is characterized by Vulcanian explosions, i.e., transient explosive events generally associated with the emission of blocks and bombs impacting an area of several hundred of meters around the vent and tephra plumes typically reaching 1–4 km (a.s.l.)^7^, producing frequent tephra fallout over inhabited areas (e.g., Kagoshima city with ~ 600,000 inhabitants). Being located in the Aira caldera, Sakurajima volcano is continuously monitored with several remote sensors such as visible and infrared imagery, but also Doppler radars^8,9,11^ and with a network of 12 PS2 (at the time of the observations)^9,50^. We carried out a 15-day measurement campaign at Sakurajima to test the LPM against tephra samples collected in trays and the monitoring network of the Sakurajima Volcano Observatory (SVO) (Fig. 1a). It is important to consider that trays were located next to the LPM, which was moved around the island. In contrast, the PS2 network is fixed. During the campaign, the activity of the Minamidake crater consisted in weak Vulcanian eruptions and almost continuous ash venting.

### The laser precipitation monitor (LPM)

The LPM is an optical disdrometer (Fig. 1b) developed by Thies Clima and originally designed to monitor the precipitation of liquid and solid hydrometeors and differentiate them based on their size/velocity signatures^26,27,51,52^. The measurement principle of optical disdrometers is based on the extinction intensity and the crossing time of falling objects through a laser sheet from which the falling velocity and the size of the objects are derived, respectively^16^. The LPM uses a laser (wavelength of 785 nm) with a theoretical measuring area *S* of 46.5 cm^2^ (22.8 × 2 cm). Basically, three operating modes are made available for the LPM: the “drop by drop”, one-minute detection and “tipping bucket simulation”^26^. In order to test the capacity of the LPM to record ash size and settling velocity, we used the “drop by drop” mode as the number of falling material was sufficiently low to allow high data transmission with the computer^26^. In the three different modes, the LPM is capable of detecting objects with sizes ranging between 0.15 and 8.00 mm and velocities between 0 and 10 m s^−1^. Therein, we also compare records of PSVDs obtained by our LPM with PS2 optical disdrometers operated by the SVO (Fig. 1a,b). The latter having a detection window for larger sizes (between 0.25 and 26.00 mm^16,47^), we consider the lower detection limit of the LPM (0.15 mm) as a strong advantage for tephra-fallout monitoring. Even though the LPM was designed to record precipitation of non-spherical particles with various densities such as hail and snow^25,52^, the efficiency of the LPM to measure the size of volcanic particles has been confirmed by the calibration tests carried out at the University of Geneva before the field campaign (more details are provided in the Supplementary Material) in addition to the validation carried out in the field (see main text). As usually done for weather precipitation, hydrometeors can be automatically discriminated by the LPM using the precipitation type characterization procedure based on the empirical relationship of Gunn & Kinzer^52,53^. However, as stated by Fehlmann et al.^52^, the exact functioning of this procedure is not clarified by the manufacturer. Moreover, such a relationship is not well suited for volcanic fallout; in fact, size, shape and density of individual particles and particle aggregates may vary significantly. Instead, we have used the drag equation of Ganser^38^, that has already been used to discriminate different types of settling tephra detected by disdrometers^7,12^.

Based on disdrometer records of PSVDs, the number density *N*(*D*) of detected objects can be computed with:

1 $$N\left(D\right)=\frac{n}{S \Delta t v\left(D\right) dD}$$

where *n* is the number of detected objects within the time interval Δ*t* (s), *S* is the laser measuring area (m^2^) that may vary from one LPM to another^43^, d*D* is the width of diameter classes (m) and *v*(*D*) is the velocity (m s^−1^) of the objects having a diameter *D* (m).

Thanks to the determination of *N*(*D*), *v*(*D*) and *D*, a sedimentation rate *SR*_*LPM*_ (kg m^−2^ s^−1^) can then be computed with the following equation:

2 $${SR}_{LPM}=\frac{\pi \rho }{6}{\int }_{{D}_{min}}^{{D}_{max}}N(D){D}^{3}v(D)\mathrm{d}D$$

where *ρ* is the density of the detected objects (kg m^−3^) as derived by water pycnometry analyses (see the following section) and d*D* is the width of size class. Indeed, to apply Eq. (2), we reshape LPM data using classes of velocity and size, as usually made for disdrometer spectra, with a very high velocity resolution d*v* = 0.05 m s^−1^ and d*D* = 0.05 mm. It is important to note that such a binning was only used for *SR* calculation and does not affect values of individual size and velocity data. Therein, *SR*_*LPM*_ will be compared to mean sedimentation rates obtained from samples collected just next to the instrument site (Fig. 1a) (see the following section).

In addition, we compare LPM estimates with the OTT optical disdrometer Parsivel^2^ (PS2). This disdrometer uses the same measurement principle than the LPM but with different specifications. In particular, it uses a laser sheet having a higher sampling area of 54 cm^2^ (18 × 3 cm) and can measure solid particles between 0.25 mm up to 26 cm with settling velocities between 0.0 and 21.4 m s^−1^. However, unlike the LPM, PS2 cannot provide information on single particles but gather them into distinct size and velocity bins (i.e., namely size and velocity classes)^8,9,12,16,25,27,47,51^. In addition of being widely operated for hydrometeor precipitation studies and monitoring, PS2 have also been used for many volcanological applications such as tephra sedimentation, dispersal modelling and calibration of other remote sensing systems^7–13,34,50^.

### Tephra sampling and analyses

In total, we collected 14 samples in plastic trays of 16.9 × 22.9 cm and 21.2 × 28.2 cm (i.e., box 1 and 2 in Table 1, respectively) next to the disdrometer site. Following the methodology of Diaz-Vecino et al.^33^, we determined the sample total masses using a 10^–3^ g-resolution weighing scale. Dividing these masses by the total duration of the fallout event provided by the LPM (Table 1) gives mean sample sedimentation rates noted *SR*_*Sample*_ (kg m^−2^ s^−1^). Diaz-Vecino et al.^33^ have worked on the same tephra samples but used camera analyses in combination with laser diffraction. Independently, in this study, we also consider 7 additional samples with respect to Diaz-Vecino et al.^33^, i.e., IV, VI, VIII, VIII-1, VIII-2, IX and XVI, to test the LPM (Tables 1 and 2).

All samples were manually sieved down to 1 Φ (i.e., 0.5 mm)^33^, with Φ = -log_2_(*D*(mm)). The remaining fractions below 0.5 mm were then analyzed based on the optical capability of the morpho-grainsizer BETTERSIZER S3 + (BETTERSIZER company: https://www.bettersizeinstruments.com/products/bettersizer-s3-plus-laser-particle-size-analyzer/; called BTS in this study) to determine both size and shape. BTS analyses consist in measuring fractions of solid particles put in a water circulating system. Once passing inside a transparent glass detection cell (CCD), two cameras with magnifications of × 0.5 and × 10 measure the size and shape of particles at a maximum rate of 10,000 particles/min and an imaging speed of 120 frames/s (User’s manual V8.0). While laser diffraction can be used in combination with the cameras, we only used camera analyses as laser data do not provide shape information on the analyzed material.

Final grain-size distributions, named GSD_BTS_, are then obtained by combining both the information of the material below and above 0.5 mm, based on the weight of each sieved fractions. To describe all individual grain-size distributions, we computed their median and sorting coefficients using the Φ scale, namely *Md*_*Φ*_ and *σ*_*Φ*_^35^. In more details, *Md*_*Φ*_ corresponds to the 50st percentile of the distributions, while *σ*_*Φ*_ is given by the difference between the 84th and the 16th percentiles divided by 2.

In terms of particle shape, we use three shape parameters: convexity, solidity and sphericity. The convexity defines the morphological roughness of tephra and corresponds to the ratio between the Convex Hull Particle perimeter and the particle perimeter (*P*)^55^. The Convex Hull defines the smallest polygon that contains all particle pixels^56^. The solidity, instead, characterizes the textural roughness of analyzed tephra and is computed with the ratio of the particle area (*A*) over its convex hull area^37,56,57^. The sphericity *Ψ*^36^ is the shape descriptor used to consider particle shapes in the Ganser model^38,40^ and calculated as *Ψ* = (4*πA*)/*P*^*2*^.

Bagheri et al.^22^ provided density estimates by water pycnometry of aggregates core between 0.064 and 1 mm that described a trend in the form *y* = 356*x* + 2720 (R^2^ of 0.95). Such a trend was similar to those derived by Miwa et al.^58^ and Hickey et al.^59^ for Sakurajima tephra^31^. In this study, we obtained water pycnometry measurements^22,60^ on sieved fractions from samples XIII and XVI to determine the density of our collected material during the measurement campaign and to evaluate whether our estimates are in agreement with previous studies (Fig. 2a). Hence, we use particle sizes, shapes and densities to filter the various falling objects (i.e., individual particles and aggregates) potentially measured by the LPM based on their size/velocity signatures. In particular, as made in previous studies involving the detection of tephra with optical disdrometers^7,12^, we used the drag equation of Ganser (1993) to model the terminal settling velocity of tephra particles with:

3 $${C}_{D}=\frac{24}{Re{K}_{1}}\left[1+0.1118{\left(Re{K}_{1}{K}_{2}\right)}^{0.6567}\right]+\left(\frac{0.4305}{1+\frac{3305}{Re{K}_{1}{K}_{2}}}\right)$$

where *C*_*D*_ is the drag coefficient which firstly depends on the shape of the particle characterized by the Stokes’ shape and the Newton’s shape factors *K*_*1*_ and *K*_*2*_, respectively in form:

4 $${K}_{1}={\left(\frac{1}{3}+\frac{2}{3}{\Psi }^{-\frac{1}{2}}\right)}^{-1}$$

and

5 $${K}_{2}={10}^{1.8148({-\mathrm{log}\Psi )}^{0.5743}}$$

in which *Ψ* is the sphericity of Riley et al.^36^ determined with the morpho-grainsizer.

Secondly, Eq. (3) depends on the Reynolds number *Re* which describes the regime of the fluid (i.e., air herein), in which the modelled particle falls:

6 $$Re=\frac{{V}_{T}D{\rho }_{a}}{\mu }$$

where *D* is the particle diameter in m, *ρ*_*a*_ and *µ* are respectively the density (kg m^−3^) and viscosity (Pa s) of air and *V*_*T*_ is the terminal settling velocity of the particle in m s^−1^.

## Supplementary Information

Supplementary Information.
