# Supplementary material for: New insights into real-time detection of tephra grainsize, settling velocity and sedimentation rate
Source: Sci Rep. 2022 Mar 17;12:4650. doi: 10.1038/s41598-022-08711-1 (PMC8931165; doi:10.1038/s41598-022-08711-1)
Supplement: Supplementary file 1 — Supplementary Information. [file 41598_2022_8711_MOESM1_ESM.pdf]

New insights into real-time detection of tephra grainsize, settling velocity and sedimentation rate

V. Freret-Lorgeril[1*], C. Bonadonna[1], Eduardo Rossi[1], A. Poulidis[2,3], M. Iguchi[2]

[1]Department of Earth Sciences, University of Geneva, 13, Rue des Maraichers, CH-1205 Geneva, Switzerland
[2]Disaster Prevention Research Institute (DPRI), Kyoto University, Kagoshima, Japan
[3]Institute of Environmental Physics (IUP), University of Bremen, Bremen, Germany
*valentin.freret-lorgeril@unige.ch

1 Summary of all variables and acronyms used in this study

**Acronym**	**Term**
BTS	BETTERSIZER
Cv	Particle Convexity
GSD	Grain-Size Distribution
GVD	Grain-Velocity Distribution
LPM	Laser Precipitation Monitor
MAT	Matsuura
PC1	Particle Cluster 1 (i.e., ash clusters[1,2])
PC2	Particle Cluster 2 (i.e., coated particles[2])
PC3	Particle Cluster 3 (i.e., cored clusters[3])
PSVD	Particle Size and Velocity Distribution
PS2	Parsivel² disdrometer
Sd	Particle Solidity
SVO	Sakurajima Volcano Observatory
TGSD	Total Grain-Size Distribution
**Symbol**	**Variable**
Δt	Time resolution of disdrometer record (s)
ρ	Particle density (kg m^{-3})
φ	Particle sphericity[4]
Φ	Particle size in Phi unit; Φ=-$\log_2$ (D(mm))
D	Particle diameter (m)
dD	Width of size class (m)
SR_{LPM}	LPM-derived sedimentation rate (kg m^{-2} s^{-1})
SR_{PS2}	PS2-derived sedimentation rate (kg m^{-2} s^{-1})
SR_{Sample}	Mean sedimentation rate of collected samples (kg m^{-2} s^{-1})
Md_{Φ}	Distribution median in phi unit[5]
σ_{Φ}	Sorting coefficient in phi unit[5]
n	Particle number
$N(D)$	Number density of particle diameter D (m^{-3} mm^{-1})
S	Measuring surface area of the disdrometer laser beam (m^2)
$v(D)$	Settling velocity of particle with diameter D (m s^{-1})

2 Grain-size data of all tephra fallout events recorded with the LPM and sampled on ground

Figure S1. Grain-size distribution of all recorded and sampled tephra fallout during the measurement campaign. Red: raw LPM data; dashed yellow: LPM data without margin fallers; Blue: LPM for individual particles; Black: BETTERSIZER raw data; Dashed Black: BETTERSIZER without particles <0.15 mm.

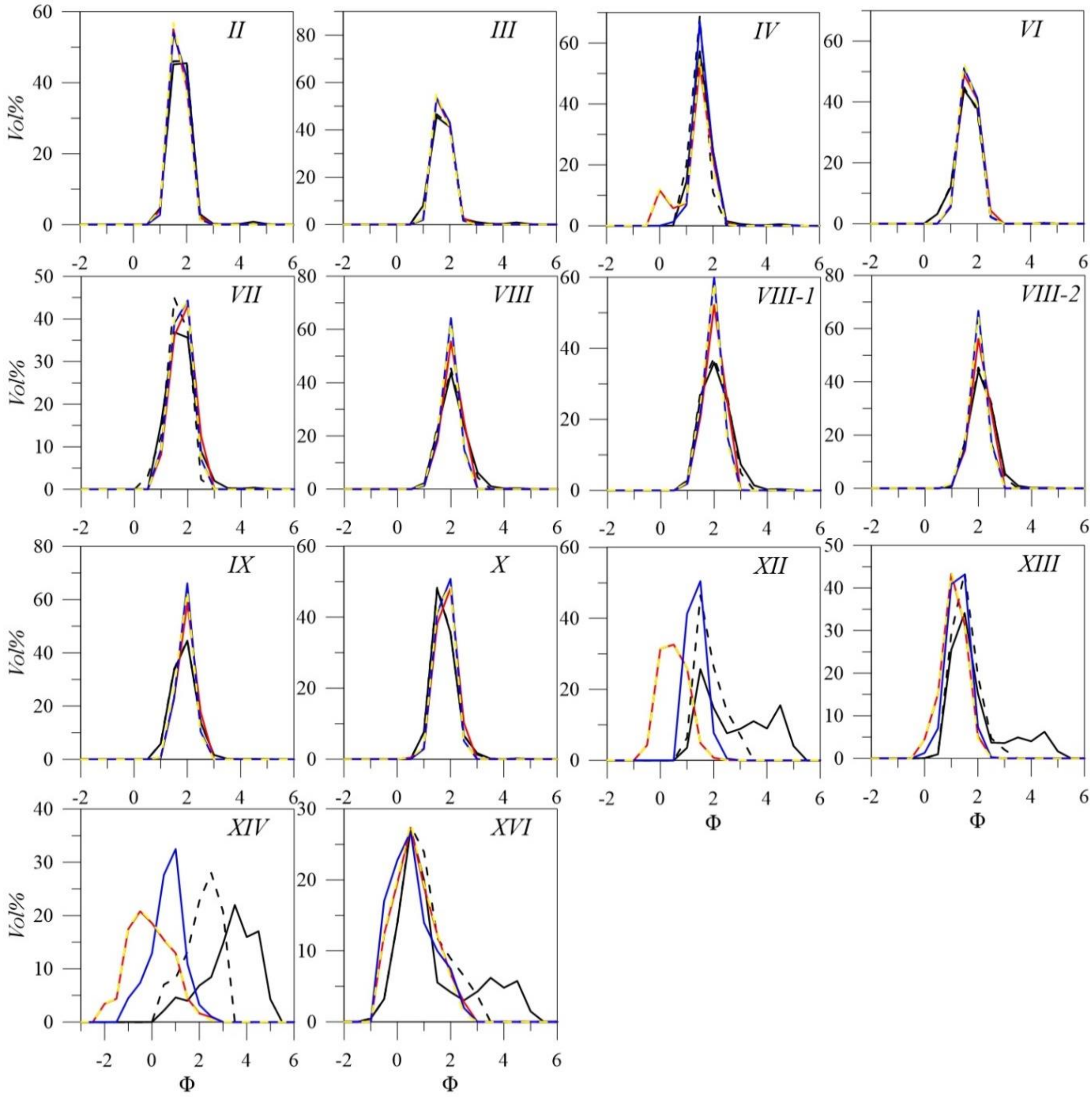

3. Grain-Size calibration of the Laser Precipitation Monitor.

Calibration tests were carried out at the university of Geneva (UNIGE) before the field campaign in order to investigate the capacity of the LPM to detect the size of ash particles. To do this, we sieved an ash sample collected from the Chaimilla tephra deposit (<3.1 ka)[6] associated with Villarrica volcano (Chile) and sieved between 710 and 1000 microns (i.e., 0.5 Φ). In total, 100 particles were randomly selected, measured with the LPM one by one and analyzed with the Bettersizer (BTS). Our results show that despite the differences of data distribution (i.e., sorting; LPM-based diameter values being more spread than BTS ones), the two devices present similar grain-size distributions between 0.6-1.6 mm and 0.9-1.3 mm, respectively, for the LPM and the BTS (Fig. S2a-c) and similar average particle diameters for the same ash population with 1.13 ± 0.20 mm for the LPM and 1.12 ± 0.10 mm for the BTS (Figure S2a,b). These tests, in addition with the validation carried out in the field (see main text) validate the capacity of the LPM to provide valuable size measurements, even for a population of non-spherical material such as volcanic ash particles.

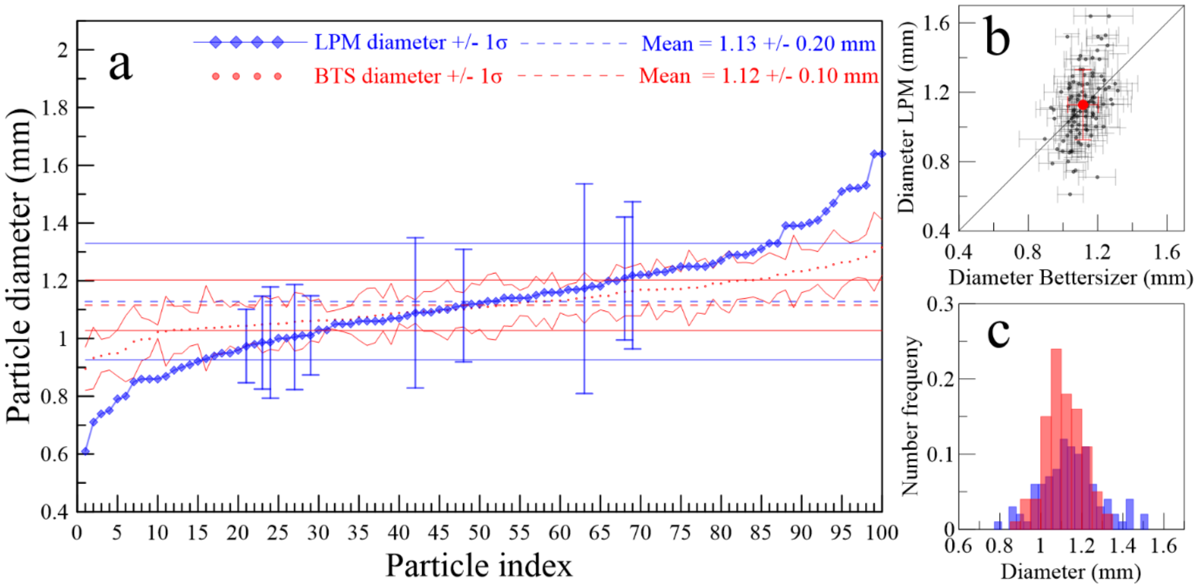

Figure S2. a) Particle diameter of 100 individual volcanic ash particles measured with the LPM (blue dots) and the Bettersizer (BTS; red dots). Straight lines indicate standard deviations (σ) on each measurement. Horizontal dashed lines indicate the average diameter of the ash population from both devices. b) Particle diameter recorded by LPM *vs* those measured with the BTS. The red dot indicates the average diameter. The black line represents the one-to-one line. c) Number frequency histograms of particle diameters from the LPM (blue) and the BTS (red).

References

1. Lane, S. J., Gilbert, J. S. & Hilton, M. The aerodynamic behaviour of volcanic aggregates. *Bull. Volcanol.* **55**, 481–488 (1993).
2. Brown, R. J., Bonadonna, C. & Durant, A. J. A review of volcanic ash aggregation. *Phys. Chem. Earth* **45-46**, 65–78. https://doi:10.1016/j.pce.2011.11.001 (2012).
3. Bagheri, G., Rossi, E., Biass, S. & Bonadonna, C. Timing and nature of volcanic particle clusters based on field and numerical investigations. *J. Volcanol. Geotherm. Res.* **327**, 520–530, doi:10.1016/j.jvolgeores.2016.09.009 (2016).
4. Riley, C. M., Rose, W. I. & Bluth, G. J. S. Quantitative shape measurements of distal volcanic ash. *J. Geophys. Res.* **108**(B10), 2504 (2003).
5. Inman, D. L. Measures for describing the size distribution of sediments. *J. Sed. Petrol.* **22**, 125–145 (1952).
6. Costantini, L., Pioli, L., Bonadonna, C., Clavero, J., Longchamp, C. A Late Holocene explosive mafic eruption of Villarrica volcano, Southern Andes: The Chaimilla deposit. *J. Volcanol. Geotherm. Res.* **200**(3–4), 143–158, doi:10.1016/j.jvolgeores.2010.12.010 (2011).
